# Readiness of health facilities to manage individuals infected with COVID-19, Uganda, June 2021

**DOI:** 10.1186/s12913-023-09380-0

**Published:** 2023-05-04

**Authors:** Patience Mwine, Immaculate Atuhaire, Sherry R. Ahirirwe, Hilda T. Nansikombi, Shaban Senyange, Sarah Elayeete, Veronicah Masanja, Alice Asio, Allan Komakech, Rose Nampeera, Edirisa J. Nsubuga, Petranilla Nakamya, Andrew Kwiringira, Stella M. Migamba, Benon Kwesiga, Daniel Kadobera, Lillian Bulage, Paul E. Okello, Sandra Nabatanzi, Fred Monje, Irene B. Kyamwine, Alex R. Ario, Julie R. Harris

**Affiliations:** 1 Uganda Public Health Fellowship Program, Kampala, Uganda; 2 Uganda National Institute of Public Health, Kampala, Uganda; 3 United States Centers for Disease Control and Prevention, Kampala, Uganda

**Keywords:** COVID-19, Pandemic, Second wave, Health facilities, Readiness, Uganda

## Abstract

**Background:**

The COVID-19 pandemic overwhelmed the capacity of health facilities globally, emphasizing the need for readiness to respond to rapid increases in cases. The first wave of COVID-19 in Uganda peaked in late 2020 and demonstrated challenges with facility readiness to manage cases. The second wave began in May 2021. In June 2021, we assessed the readiness of health facilities in Uganda to manage the second wave of COVID-19.

**Methods:**

Referral hospitals managed severe COVID-19 patients, while lower-level health facilities screened, isolated, and managed mild cases. We assessed 17 of 20 referral hospitals in Uganda and 71 of 3,107 lower-level health facilities, selected using multistage sampling. We interviewed health facility heads in person about case management, coordination and communication and reporting, and preparation for the surge of COVID-19 during first and the start of the second waves of COVID-19, inspected COVID-19 treatment units (CTUs) and other service delivery points. We used an observational checklist to evaluate capacity in infection prevention, medicines, personal protective equipment (PPE), and CTU surge capacity. We used the “ReadyScore” criteria to classify readiness levels as > 80% (‘ready’), 40–80% (‘work to do’), and < 40% (‘not ready’) and tailored the assessments to the health facility level. Scores for the lower-level health facilities were weighted to approximate representativeness for their health facility type in Uganda.

**Results:**

The median (interquartile range (IQR)) readiness scores were: 39% (IQR: 30, 51%) for all health facilities, 63% (IQR: 56, 75%) for referral hospitals, and 32% (IQR: 24, 37%) for lower-level facilities. Of 17 referral facilities, two (12%) were ‘ready’ and 15 (88%) were in the “work to do” category. Fourteen (82%) had an inadequate supply of medicines, 12 (71%) lacked adequate supply of oxygen, and 11 (65%) lacked space to expand their CTU. Fifty-five (77%) lower-level health facilities were “not ready,” and 16 (23%) were in the “work to do” category. Seventy (99%) lower-level health facilities lacked medicines, 65 (92%) lacked PPE, and 53 (73%) lacked an emergency plan for COVID-19.

**Conclusion:**

Few health facilities were ready to manage the second wave of COVID-19 in Uganda during June 2021. Significant gaps existed for essential medicines, PPE, oxygen, and space to expand CTUs. The Uganda Ministry of Health utilized our findings to set up additional COVID-19 wards in hospitals and deliver medicines and PPE to referral hospitals. Adequate readiness for future waves of COVID-19 requires additional support and action in Uganda.

## Introduction

Ensuring the readiness of health facilities (hospitals, health centers, and clinics) to respond during public health emergencies is essential to effective epidemic management [1]. Health facility readiness is defined as a combination of the presence of appropriate infrastructure and amenities, basic supplies and equipment, laboratory tests, medicines and commodities, and trained health professionals [2]. While many types of resources are required for effective response, even countries with highly-resourced health care systems faced challenges with adequate readiness during the COVID-19 pandemic [3, 4].

In February 2020, the World Health Organisation (WHO) released a COVID-19 strategic response preparedness plan to guide health facilities in preparing for COVID-19 outbreaks [4]. The plan’s key pillars included coordination of the response across different administrative levels, risk communication, infection prevention and control, logistics, and medicines, ensuring continuity of other health services, and planning for surge capacity [1]. In line with these pillars, in May 2020 Uganda’s Ministry of Health (MoH) equipped National Referral Hospital (NRH) and Regional Referral Hospitals (RRHs) with trained health care workers and a COVID-19 treatment unit and provided extra supplies of medicines and personal protective equipment (PPE) [5]. Lower-level health facility health workers were trained to screen, identify, and manage mild cases, and to refer severe COVID-19 cases to referral health facilities.

From March 21, 2020, when the first case was reported, through approximately August 2020, Uganda registered few COVID-19 cases, mostly among travellers and their contacts [6]. However, community transmissions led to a rapid increase in cases that started in August 2020 and peaked around December 2020. By January 2021, at the end of the first wave, 39,847 confirmed COVID-19 cases and 324 deaths had been recorded in Uganda [7]. During the peak of the first wave, health facilities faced major challenges in providing adequate care for COVID-19 patients, including lack of appropriate health facility infrastructure such as oxygen cylinders and patient beds, lack of sufficient trained health care workers, and insufficient supplies of PPE [8]. After a respite between waves of a few months, the second wave of COVID-19 began in May 2021 [7].

The second wave of COVID-19 in Uganda was driven primarily by the SARS-CoV-2 Delta variant, which was concurrently causing massive outbreaks in many other countries, including neighbouring Kenya [9]. Despite efforts to improve health facility readiness after the first wave, including via the installation of ICU beds and ventilators at Mulago National Referral Hospital and some of the regional referral hospitals, it was unclear how ready health facilities were for the second wave [10]. We assessed health facility readiness to manage the second wave of COVID-19 in Uganda and identified areas for improvement to strengthen capacity for future waves of COVID-19 cases.

## Methods

### Study setting

As of November 2018, Uganda had a total of 6,937 health facilities, including public, private not-for-profit, and private for-profit facilities [11]. Of these, 3,133 (45%) were public health facilities, which provide free health care to the general population with support from the government and partners. Public health facilities are classified into Health Centers Level Two (HC II; the most basic health level) through Four (HC IV), general hospitals, regional referral hospitals (RRH), and national referral hospitals (NRH). At the start of the COVID-19 pandemic, the MoH established COVID-19 treatment units (CTUs) in 3 NRH and 14 RRH (of the total 20 referral hospitals) in Uganda. These CTUs were equipped with oxygen cylinders, beds, and medicines for managing COVID-19 (such as azithromycin, zinc and Vitamin C) [12]. New and existing health care workers at the facilities were also trained on COVID-19 case management and PPE appropriate for COVID-19 was distributed to facilities. A single advanced-level CTU with advanced life support machines was set up at Mulago National Referral Hospital (MNRH) to provide care to the most critically ill COVID-19 patients.

### Site selection

#### Health facilities

We selected all 17 referral health facilities, including three national referral hospitals (NRH) and 14 regional referral hospitals (RRH) that were managing COVID-19. We selected 71 lower-level health facilities using multistage sampling. First, we randomly divided the country into seven subregions and selected two districts from each: one with and the other without a referral health facility. From each district, we listed all the health facilities and randomly selected one general hospital (GH), one health centre IV (HC IV), two health centres III (HC III), and two health centres II (HC II).

#### Health care workers

We conducted in-person interviews with each health facility head or CTU visited about COVID-19 response challenges in their health facilities.

### Study variables and data collection

We interviewed heads of health facilities using a structured questionnaire. We obtained information on the first and the start of the second waves of COVID-19 in relation to case management, coordination and communication and reporting, and preparation for the surge of COVID-19. We conducted on-site inspection of the CTUs and other service delivery points in the health facility using a readiness assessment tool developed by the US Centers for Disease Control and Prevention (CDC) for non-US health care settings and revised to suit Uganda’s situation [13]. We assessed facility-level response coordination by checking for documentation of health facility meeting minutes on COVID-19 response, availability of an emergency response plan, and communication systems in place necessary for coordination and reporting of COVID-19 cases to the MoH. To verify if the health workers were trained, we asked the respondents to describe the processes where appropriate, which we then compared with the MoH standards. We made a physical count of the appropriate PPE and medicines for the management of COVID-19 and compared the counts to the average monthly consumption of individual health facilities. In addition to these, we observed for oxygen equipment (oxygen cylinders, oxygen concentrators, Oxygen plants and masks) and space for CTU expansion at referral facilities in case of a surge of COVID-19 patients. We checked for documentation of training and mentorship of health facility staff on COVID-19 and standard operating procedures for infection prevention. We also observed service delivery points, checked for infection prevention measures and the presence and functioning of triage systems. Both the questionnaire and the checklist were in an electronic form prepared using KoBoToolbox [14].

### Data analysis

We imported clean data into EpiInfo version 7 for analysis. We assigned a value of one to a “Yes” response and a zero to a “No” response and computed readiness scores for each health facility as the proportion of the responses that were “Yes”. We determined a facility’s level of readiness using the “ReadyScore” criteria [15]. The criteria were developed by Resolve to Save Lives and based on existing data from the Joint External Evaluation (JEE) by the World Health Organization after the 2014 Ebola epidemic [16]. These criteria were designed to help countries determine their capacity to prevent, stop, or control an epidemic, and identify life-threatening preparedness gaps and close them. The ReadyScore criteria scale ranges from 0 to 100 and there are five levels of readiness including, unknown, in progress, “not ready” if the score is (< 40%), “work to do” for (40–80%) scores and ready if (> 80%) scores. We used these criteria to categorise the individual facility percentage scores. The classification of readiness of the health facilities was specific to the level of the health facility. We considered 59 questions for the lower-level health facilities related to coordination, communication, reporting, supplies, training, triage, and evaluation of COVID-19 suspects. In addition to these questions, we assessed the provision of care for referral to the health facilities, the monitoring of health care workers and inpatients, and the preparation for a surge of COVID-19 cases to make a total of 71 questions for the other facility levels.

We used weighted analysis for lower-level health facilities, basing the weights on the strata (districts with and without referral hospitals) and the representation of the selected facilities, by facility level, within each stratum. For example, weights for HC II in districts with referral hospitals were calculated based on the total number of HC II and the number of HC II selected within those districts. We used complex sample frequencies in Epi-Info to obtain percentages of each variable. We used QGIS software to map the geographical distribution of the health facilities visited.

## Results

### Characteristics of assessed health facilities

**Fig. 1:**
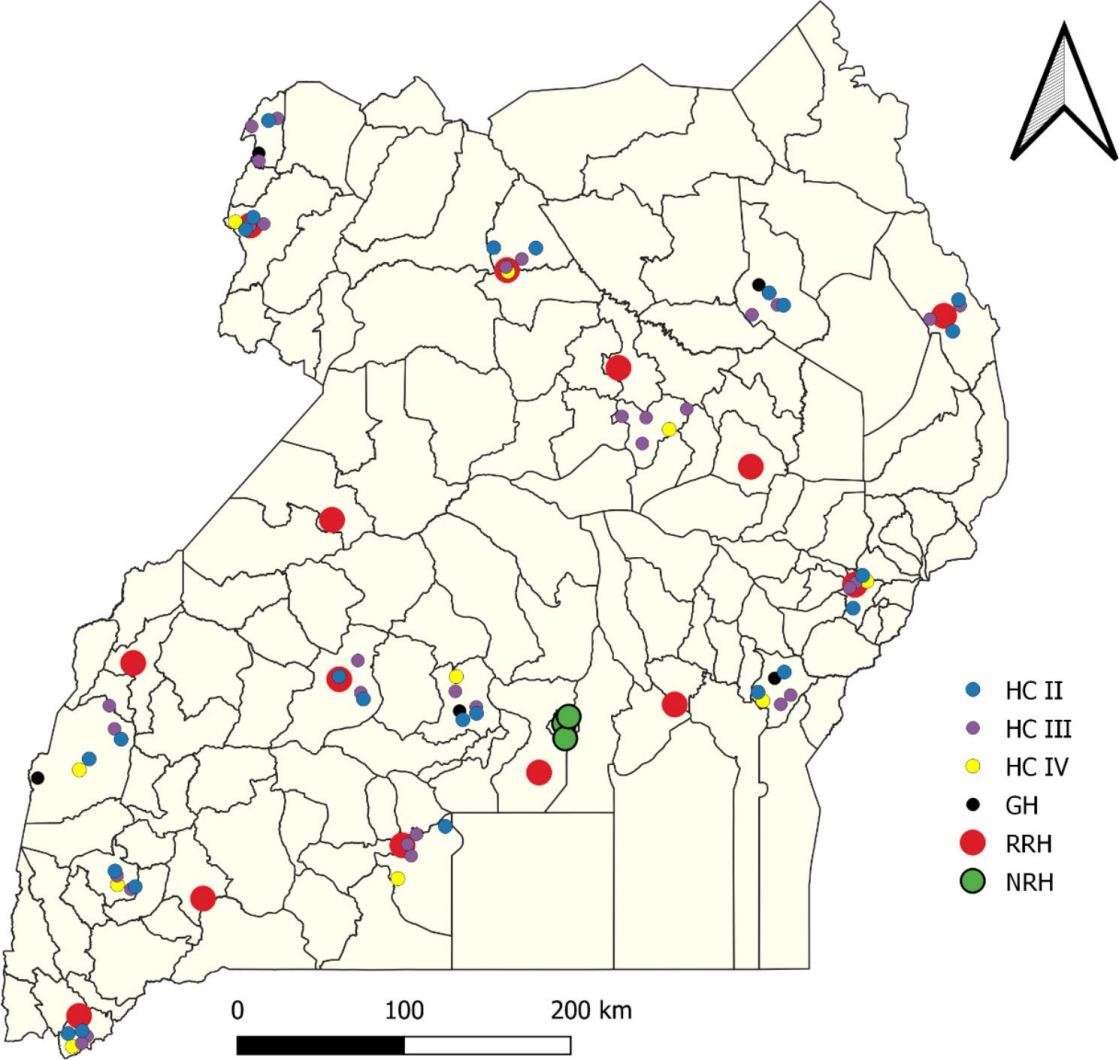
Location of health facilities evaluated for COVID-19 readiness, Uganda, June 2021 *NRH-National referral hospital; RRH- Regional referral hospital; GH- General hospital; HC-Health center

The 88 health facilities assessed were widely distributed across the country (Fig. 1). At the time of the assessment, all 17 referral health facilities were managing COVID-19 patients, and no lower-level health facilities had COVID-19 patients isolated.

### Health facility readiness scores

**Fig. 2:**
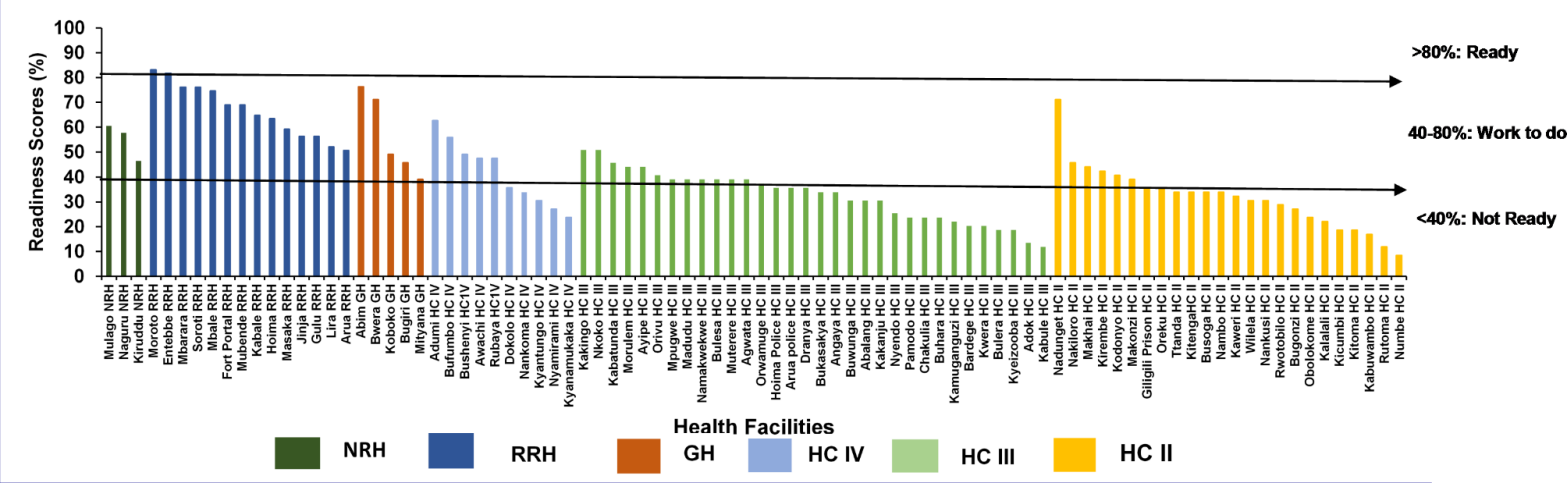
Health facility readiness to manage COVID-19 cases during the second wave in Uganda, June 2021 *NRH-National referral hospital; RRH- Regional referral hospital; GH- General hospital; HC-Health center

The overall median (interquartile range; IQR) readiness score for all health facilities was 39% (IQR: 27, 51%). The median readiness score in referral facilities was 63% (IQR: 56, 75%), while the weighted median score for lower-level facilities was 32% (IQR: 24, 37%). Of the 17 referral facilities, only two (12%), both regional referral hospitals, were “ready”, while 15 (88%) were in the “work to do” category. Fifty-five (77%) lower-level health facilities were in the “not ready” category (Table 1). The health facility readiness decreased with decreasing level of the facility; most of the lower-level health facilities were not ready (Fig. 2).

**Table 1:** Health facility readiness to manage the second wave of COVID-19 based on Resolve “ReadyScore” criteria, Uganda, June 2021

Level of Health facility (n)	“Not ready” (n, %)		“Work to do” (n, %)		“Ready” (n, %)	
National Referral Hospitals (n = 3)	0	(0)	3	(100)	0	(0)
Regional Referral Hospital (n = 14)	0	(0)	12	(86)	2	(14)
General Hospital (n = 5)	1	(20)	4	(80)	0	(0)
Health Center IV (n = 10)	5	(50)	5	(50)	0	(0)
Health Centre III (n = 32)	28	(87)	4	(13)	0	(0)
Health Center II (n = 24)	21	(88)	3	(12)	0	(0)

### Coordination, reporting, and preparation for the surge

Referral health facilities scored well in the coordination component of the assessment, with 100% having an IPC focal person and 82% having an emergency response plan describing the arrangement, responsibilities, and activities to enable the hospital to function adequately in the COVID-19 response. Most referral health facility heads/CTU heads (14; 82%) knew their maximum capacity in the event of a surge of COVID-19 cases. However, 11 (65%) reported that they could not identify additional space to accommodate expanding numbers of COVID-19 patients if needed, and 12 (71%) did not include in their plans the option to stop non-essential services in case of an overwhelming surge of COVID-19 cases (Table 2).

**Table 2:** Comparison of coordination, communication, and reporting systems among referral and lower-level health facilities during the second wave of COVID-19, Uganda, June 2021

	Referral Health Facilities (n = 17)				Lower-Level Health Facilities* (n = 71)			
	Yes		No		Yes		No	
	n	%	n	%	n	%	n	%
**Coordination**								
Facility has an IPC focal person in place	17	(100)	0	(0)	63	(82)	8	(18)
IPC team participates in emergency committee meetings	17	(100)	0	(0)	17	(20)	54	(80)
The facility has an emergency response plan for COVID-19	14	(82)	3	(18)	18	(27)	53	(73)
The facility has an emergency committee that meets weekly	13	(76)	4	(24)	12	(14)	59	(86)
**Communication and reporting**								
Facility has a dedicated person to report suspected or confirmed COVID-19 cases	17	(100)	0	(0)	42	(52)	29	(48)
HCW know the referral system for suspected or confirmed COVID-19 cases	17	(100)	0	(0)	56	(74)	15	(26)
The facility has a phone number for people to report suspected cases	16	(94)	1	(6)	55	(71)	16	(29)
HCW understand reporting levels of suspected or confirmed COVID-19 cases	16	(94)	1	(6)	57	(74)	14	(26)
**Preparation for the surge in CTU**								
The HCW know the maximum capacity of the CTU	14	(82)	3	(18)	NA	-	NA	-
The facility has developed a plan to move non-critical patients in case of a surge	12	(71)	5	(29)	NA	-	NA	-
The facility has estimated consumption rates for critical supplies such as PPEs, oxygen, and medicines	11	(65)	6	(35)	NA	-	NA	-
The facility was able to identify additional space to expand the number of COVID-19 patients	6	(35)	11	(65)	NA	-	NA	-
The facility has included in its plan the option to stop non-essential services	5	(29)	12	(71)	NA	-	NA	-

***IPC-Infection Prevention Control; CTU-COVID-19 Treatment Unit; HCW-Health Care Workers; PPE-Personal Protective Equipment***

*Percentages for the lower-level health facilities are the weighted scores

Comparatively, lower-level health facilities were poorly prepared. Fifty-three (73%) lower-level health facilities lacked emergency response plans for COVID-19. Communication and reporting were poor in lower-level health facilities; 29 (48%) lacked personnel designated to report suspected or confirmed cases of COVID-19 (Table 2).

### Training and triage

In all 17 referral health facilities, all health workers had received at least training to recognize COVID-19 symptoms. However, 26 (35%) lower-level health facilities reported that their health workers did not receive COVID-19 training. Triage for respiratory patients was lacking in lower-level health facilities and some referral health facilities; in addition, 59 (82%) of the lower-level health facilities and 8 (47%) referral facilities lacked a physical barrier to separate health workers and patients during the patient review. Fifty-four (76%) lower-level health facilities and two (29%) referral hospitals lacked areas to isolate patients with acute respiratory symptoms (Table 3).

**Table 3:** Comparison of training and triage systems among referral and lower-level health facilities during the second wave of COVID-19, Uganda, June 2021

	Referral Health Facilities (n = 17)				Lower-Level Health Facilities* (n = 71)			
	Yes		No		Yes		No	
	n	%	n	%	n	%	n	%
**Training**								
All HCWs were trained at least once to recognize COVID-19 symptoms	17	(100)	0	(0)	45	(65)	26	(35)
HCWs managing COVID-19 trained at least once in transmission-based precautions	16	(94)	1	(6)	NA	-	NA	-
Cleaners trained in safe cleaning CTU/isolation units	14	(82)	3	(18)	NA	-	NA	-
**Triage and evaluation of suspected COVID-19 cases**								
Functional hand hygiene available near the registration desk and respiratory waiting area	17	(100)	0	(0)	50	(68)	21	(32)
Access to PPE by HCW during patient examination	13	(76)	4	(24)	24	(27)	47	(73)
Plans for the safe transfer of patients with suspected or confirmed COVID-19 identified	13	(76)	4	(24)	36	(51)	35	(49)
The facility has a separate “respiratory waiting area” to isolate patients with respiratory symptoms	12	(71)	5	(29)	17	(24)	54	(76)
Benches, chairs, or other seating in the respiratory waiting area are separated by at least 1 m	12	(71)	5	(29)	15	(21)	56	(79)
A separate room for conducting physical evaluations of other patients	11	(65)	6	(35)	10	(15)	61	(85)
Have ways for patients with respiratory symptoms to communicate to the COVID-19 focal person before presenting to the facility	10	(59)	7	(41)	14	(18)	57	(82)
COVID-19 triage forms and flow charts available	9	(53)	8	(47)	12	(18)	59	(82)
The facility has physical barriers to separate health workers and patients during patient review	9	(53)	8	(47)	14	(18)	59	(82)
The facility has signs to direct patients with respiratory symptoms to the respiratory waiting area	8	(47)	9	(53)	9	(11)	62	(89)
The facility has increased staff dedicated to triage for COVID-19	7	(41)	10	(59)	6	(9)	65	91)
Dedicated toilets are available for patients in the respiratory waiting area	4	(24)	13	(76)	6	(10)	65	(90)

***PPE-Personal Protective Equipment; HCW-Health Care Worker; CTU-COVID-19 Treatment Unit***

*The percentages for the lower-level health facilities are the weighted scores

### Medicines and personal protective equipment supply

More heads at referral facility CTUs (16; 94%) than heads at lower-level health facilities (46; 65%) knew how to estimate the critical PPE supply consumption rate. In relation to the number of COVID-19 patients admitted at the time of assessment, 14 (82%) referral health facilities lacked essential medicines, nine (53%) lacked adequate PPE appropriate for COVID-19, and 12 (71%) did not have adequate oxygen supply and cylinders. Among lower-level facilities, all but one (70; 99%) lacked medicines, and most (65; 92%) had inadequate PPE supplies (Table 4).

**Table 4:** Comparison of availability of essential medicines and personal protective equipment among referral and lower-level health facilities during the second wave of COVID-19, Uganda, June 2021

	Referral Health Facilities (n = 17)				Lower-Level Health Facilities (n = 71)			
	Yes		**No**		**Yes**		**No**	
	**n**	**%**	n	**%**	**n**	**%**	**n**	**%**
**Essential medicines**								
Isolation spaces and/or isolation units	16	(94)	1	(6)	9	(12)	62	(88)
HDU/ICU	13	(76)	4	(24)	NA	-	NA	-
Vitamin C	6	(35)	11	(65)	6	(7)	65	(93)
Adequate oxygen Supply and oxygen cylinders	5	(29)	12	(71)	NA	-	NA	-
Zinc	4	(24)	13	(76)	8	(9)	63	(91)
Adequate medicines for the management of COVID-19	3	(18)	14	(82)	1	(1)	70	(99)
Dexamethasone	3	(18)	14	(82)	6	(2)	65	(98)
Azithromycin	2	(12)	15	(88)	0	(0)	71	(100)
Clexane	2	(12)	15	(88)	NA	-	NA	-
Ramdesivir	0	(0)	17	(100)	NA	-	NA	-
**PPE supplies**								
Available focal person to manage critical IPC supplies	17	(100)	0	(0)	59	(80)	12	(20)
Facility leadership knows how to request additional supplies	17	(100)	0	(0)	56	(79)	15	(21)
Consumption rate (per week) for critical supplies estimated	16	(94)	1	(6)	46	(65)	25	(35)
Monthly inventory of PPE supply done at least once a month	16	(94)	1	(6)	45	(58)	26	(42)
Inventory of PPE supplies done in the past seven days	9	(53)	8	(47)	14	(21)	57	(79)
Adequate PPE for the management of COVID-19 available	8	(47)	9	(53)	6	(8)	65	(92)
**The facility has the following PPE supplies in stock**								
Aprons	13	(76)	4	(24)	4	(2)	67	(98)
Eye protection (face shields or goggles)	13	(76)	4	(23)	5	(3)	66	(97)
Gowns	12	(71)	5	(29)	4	(2)	67	(98)
Alcohol-based hand rub	12	(71)	5	(29)	10	(12)	61	(88)
N95, or equivalent respirators	10	(59)	7	(41)	11	(12)	60	(88)
Hospital-grade disinfectants (Sodium hypochlorite)	10	(59)	7	(41)	7	(7)	64	(93)
Soap	9	(53)	8	(47)	35	(48)	36	(52)
Buckets	8	(47)	9	(53)	5	(5)	66	(95)
Surgical face masks	5	(29)	12	(71)	12	(14)	59	(86)

***HDU-High Dependence Unit; ICU-Intensive Care Unit; PPE Personal Protective Equipment; IPC-Infection Prevention and Control***

*The percentages for the lower-level health facilities are the weighted scores

## Discussion

In May 2021, early during the second wave of COVID-19 in Uganda, the readiness of most health facilities to manage COVID-19 cases was poor. Triage systems and supplies of medicines, PPE, and oxygen for the management of COVID-19 were lacking in many health facilities. Beyond this, few referral facilities were able to expand their COVID-19 patient capacity in the event of a surge.

The overall median readiness score for health facilities (39%) was below the recommended target score of at least 80% [15]. Scores were lower in the lower-level health facilities than in the referral health facilities. The primary driving force behind the disparity in scores was differences in the availability of PPE and essential medicine supplies between the facility levels. Shortages of PPE are associated with increased risk of COVID-19 infections among health care workers [17]. Unfortunately, infected health workers also become the source of infections to the patients, families, and communities where they live [18]. PPE shortages were also noted during the COVID-19 pandemic in other studies in Uganda [19], as well as in other countries including in high-income countries, driven by the high demand during COVID-19 waves [20]. However, the disparity in PPE shortages between higher-level and lower-level facilities in Uganda may also be partially attributable to the differences in the supply delivery system used in the country. All government-funded health facilities receive medicines and PPE quarterly from the National Medical Stores (NMS). A ‘pull’ system is used to supply drugs and consumables to referral facilities and Health Centers IV; these health facilities make their quarterly orders based on average monthly consumption rates and these are filled by NMS. In contrast, lower-level health facilities rely on a ‘push’ inventory control system, in which the NMS forecasts the quantity of drugs and PPE and delivers them to the lower health facilities [21]. For these facilities, inaccuracies in predictions and an inability to control their own supply can potentially result in stockouts of drugs and supplies [22]. Using facility-specific data for forecasting could potentially alleviate this issue. Alternately, a transition to the “pull” system could be useful for lower-level health facilities to allow ordering based on needs and possibly prevent early stockouts.

We observed that most health facilities lacked triaging systems and triage staffing. At the beginning of the pandemic, WHO recommended that all health facilities have COVID-19 triaging stations, irrespective of the health facility level, to improve the identification of possible cases before they entered facilities and potentially spread infection [23]. A triage system involves screening all patients for COVID-19 symptoms, isolation of patients with symptoms, and ensuring infection prevention measures such as the strict wearing of masks and physical distancing to limit transmission of COVID-19 [24]. It is fairly inexpensive and, when done correctly, can reduce spread of COVID-19 within health facilities [25]. The MoH provided guidelines on management of COVID-19, including triage, in April 2020 [26], and nationwide training was conducted. However, the training was primarily provided to referral facilities and high-volume lower-level health facilities; from our assessment, 35% of lower-level health facilities reported that they did not receive the training. Thus, many facilities were not trained in the importance or setup of triage, which likely contributed to the poor triage scores across lower-level facilities. Understaffing may also have contributed to this problem. Uganda has a shortage of health care workers, with roughly one health professional for every 1,000 people in 2019 [27]. In addition, a February 2021 study in five Ugandan hospitals showed that most nurses who were managing COVID-19 patients reported increased workload and understaffing [28]. Similar challenges in increased workload and staff shortages were reported by several countries during the pandemic [27–29]. Some countries invested in the use of digital methods such as artificial intelligence [30] web-based self-triage [31] to triage COVID-19 patients; studies revealed they had a substantial effect in controlling the spread and transmission of COVID-19 between patients and healthcare workers [32].

Two-thirds of health facilities admitting COVID-19 patients in our study lacked extra space for admitting more COVID-19 patients in case of a surge. In other countries, such challenges led to some health facilities utilising other wards, such as emergency departments, to manage COVID-19 patients, while others turned away COVID-19 patients [33]. Similarly, we observed some facilities using emergency department space or other wards to deal with patient overflow. This lack of surge space was also an issue during the first wave, which led to the adoption of the home-based care strategy in Uganda for all but the most severely ill patients [34]. Starting in September 2020, a national football stadium was also transformed into a COVID-19 treatment unit to manage COVID-19 patients with mild disease during the surges [35].

According to WHO, at least 80% of COVID-19 cases are mild and can be managed as outpatients [24]. However, patients who develop critical symptoms may require hospital admission and oxygen therapy to reduce mortality [36]. We observed that most health facilities managing COVID-19 patients did not have an adequate supply of oxygen. This, too, was reported in several African countries during the peaks of COVID-19 cases [37]. These shortages have been attributed to increased demand and high consumption of oxygen by COVID-19 patients [37–39]; according to WHO, when comparing oxygen consumption, COVID-19 patients require three times more oxygen than non-COVID-19 patients [39, 40]. The high demand for oxygen during peaks of COVID-19 waves led to the malfunctioning of oxygen plants initially installed in Uganda’s referral facilities [41, 42]. Beyond this, there were inadequate numbers of oxygen cylinders in relation to rising COVID-19 cases [37]. To solve oxygen shortages during and after the second wave, the MoH procured more oxygen cylinders (43,44), other companies started producing oxygen in-country [42], and a large oxygen production plant was installed at the national hospital [43].

There were some limitations in the assessment. Responders may have been biased to seem more prepared than they were, or even less prepared in order to advocate for additional support such as training staff. In addition, due to variabilities in training and supply of essential medicines and PPE across the health facilities, it was challenging to attribute the gaps in specific facilities to specific causes. We were un able to reach the anticipated number of lower level health facilities due to some districts missing particular levels of health facilities. Nevertheless, we subjected our calculations to weighting to cater for the differences in the health facilities.

## Conclusion

Few health facilities were ready to manage COVID-19, necessitating additional support from the Government of Uganda and other supporting partners. Major gaps were in essential drugs, PPEs, and oxygen, and the capacity to admit more COVID-19 patients. We presented our findings to the MoH, and the incident management team utilized them to support the health facilities in the response. In addition, the findings from the survey were utilised by the MoH to plan for the possible future COVID-19 case surges: for example, more oxygen cylinders were procured for the health facilities to prevent oxygen shortages during other surges. The National Medical Stores made an emergency supply of medicines and personal protective equipment to the under-equipped referral hospitals. Also, an isolation ward was created at Kiruddu National Referral Hospital to separate COVID-19 patients from those with other medical conditions. Infection prevention and control were strengthened and respiratory areas were created in health facilities across the country.

## Data Availability

The datasets upon which our findings are based belong to the Uganda Public Health Fellowship Program, Ministry of Health, Uganda. For confidentiality reasons, the datasets are not publicly available. However, the data sets can be made available upon reasonable request from the corresponding author (pmwine@musph.ac.ug) and with permission from the Uganda Public Health Fellowship Program.

## List of Abbreviations

COVID-19: Corona Virus Disease
IQR: Inter-quartile Range
MoH: Ministry of Health
GH: General Hospital
HC: Health Center
RRH: Regional Referral Hospital
WHO: World Health Organisation
CTU: COVID-19 Treatment Unit
